# Targeting autocrine amphiregulin robustly and reproducibly inhibits ovarian cancer in a syngeneic model: roles for wildtype p53

**DOI:** 10.1038/s41388-021-01784-8

**Published:** 2021-04-30

**Authors:** Moshit Lindzen, Soma Ghosh, Ashish Noronha, Diana Drago, Nishanth Belugali Nataraj, Orith Leitner, Silvia Carvalho, Einav Zmora, Stav Sapoznik, Keren Bahar Shany, Keren Levanon, Dan Aderka, Belinda Sánchez Ramírez, Maik Dahlhoff, Iain McNeish, Yosef Yarden

**Affiliations:** 1 Departments of Biological Regulation, Weizmann Institute of Science, Rehovot, Israel; 2 Biological Services, Weizmann Institute of Science, Rehovot, Israel; 3 Sheba Cancer Research Centre, Chaim Sheba Medical Center and Sackler Faculty of Medicine, Tel Aviv University, Ramat Aviv, Israel; 4 Direction of Immunology and Immunotherapy, Center for Molecular Immunology, Havana, Cuba; 5 Institute of In Vivo and In Vitro Models, University of Veterinary Medicine Vienna, Vienna, Austria; 6 Imperial College and Hammersmith Hospital, London, UK

**Keywords:** Drug development, Ovarian cancer

## Abstract

Ovarian cancer (OvCA) remains one of the most devastating malignancies, but treatment options are still limited. We report that amphiregulin (AREG) can serve as an effective and safe pharmacological target in a syngeneic murine model. AREG is highly abundant in abdominal fluids of patients with advanced OvCa. In immunocompetent animals, depletion or overexpression of AREG respectively prolonged or shortened animal survival. A new antibody we generated in AREG-knockout mice recognized murine AREG and reproducibly prolonged animal survival in the syngeneic model. The underlying mechanism likely involves binding of wildtype p53 to *AREG*’s promoter and autocrine activation of the epidermal growth factor receptor (EGFR), a step blocked by the antibody. Accordingly, depletion of p53 downregulated AREG secretion and conferred tolerance, whereas blocking an adaptive process involving CXCL1, which transactivates EGFR, might increase therapeutic efficacy. Consistent with these observations, analysis of OvCa patients revealed that high AREG correlates with poor prognosis of patients expressing wildtype *TP53*. In conclusion, clinical tests of the novel antibody are warranted; high AREG, normal *TP53*, and reduced CXCL1 activity might identify patients with OvCa who may derive therapeutic benefit.

## Introduction

The high mortality of ovarian cancer (OvCa), which is directly associated with disseminated peritoneal metastasis, is attributed to delayed diagnosis and the fact that treatment has only moderately changed since the late 1990s [1]. Thus, other than an antibody to the vascular endothelial growth factor (VEGF) and poly(ADP-ribose) polymerase inhibitors, no molecular targeted therapies have so far been approved for OvCa. Viewed from a genetic perspective, this relates to frequent activation of undruggable tumor suppressors like p53, and scarcity of druggable activated oncogenes [2]. Because the EGF and neuregulin (NRG) families of growth factors act as major mitogens of the ovarian epithelium, they might present an important node of intervention. In line with this, a screen for potential “addicting oncogenes” using RNA interference found that an autocrine loop involving NRG1 operates in a subset of OvCa [3]. While none of the NRGs binds with epidermal growth factor receptor (EGFR), amphiregulin (AREG), epiregulin (EREG), and epigen bind to EGFR with relatively low affinity [4]. We previously identified AREG as an abundant and essential growth factor of OvCa [5]. Notably, several stimuli can regulate production of AREG. The list includes chronic inflammation [6], treatment with chemotherapy [5], and expression of either the wildtype [7] or the mutant form of p53 [8].

Another function of AREG was revealed by studies that addressed host resistance and immune tolerance [9]. Since AREG is expressed by multiple populations of activated immune cells, such as tissue-resident regulatory T cells [10], it promotes restoration of tissue integrity following damage associated with inflammation [11, 12]. Therefore, we hypothesized that intercepting AREG in the context of OvCa would inhibit tumor progression by blocking autocrine signaling, as well as by preventing local suppression of the immune system. Testing this prediction entailed an immunocompetent mouse model and generation of an antibody able to recognize the murine form of AREG. Here we describe such an antibody and its effects on the most commonly used syngeneic OvCa model, ID8 [13]. Importantly, we conclude that autocrine, rather than immune cell-derived AREG, is essential for tumor progression. Furthermore, blocking AREG emerges as a safe and effective treatment, and the status of p53 might serve as a predictive biomarker.

## Results

### Because AREG, like VEGF, is highly abundant in patients’ ascites fluids we generated an antibody that can block both human and murine AREGs

**Fig. 1:**
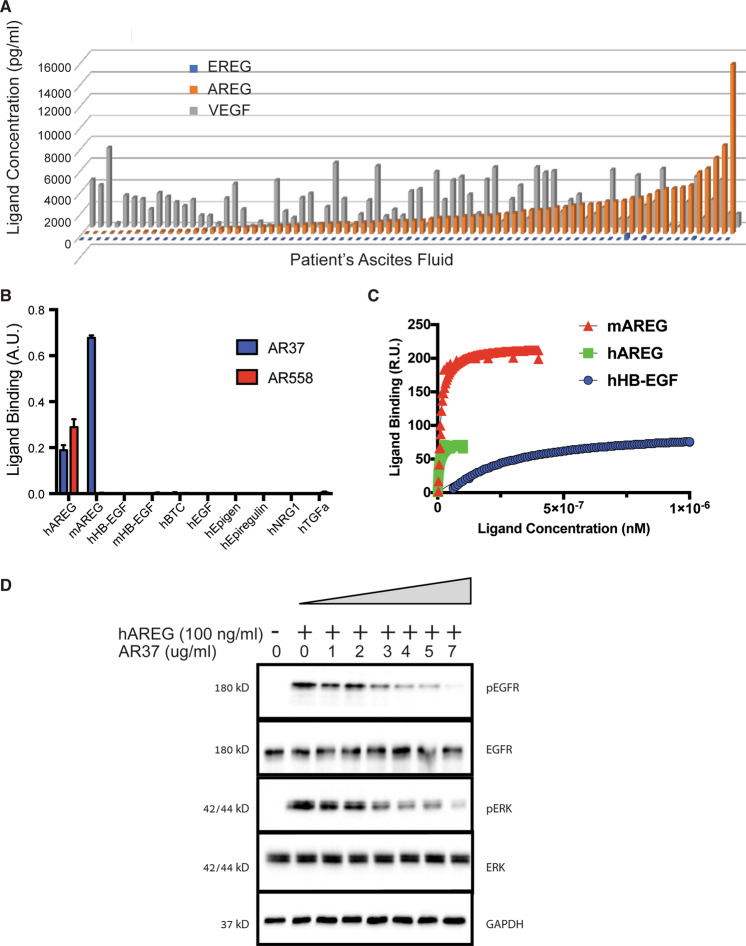
In similarity to VEGF, AREG is highly abundant in patients’ ascites fluids; a new anti-AREG antibody recognizes murine AREG. **A** Histograms presenting the abundance of VEGF, epiregulin (EREG), and AREG in ascites fluids from patients with OvCa, as determined using ELISA kits (from R&D Systems). **B** Trays of 96-wells were incubated overnight with the indicated ligand-containing solutions. Thereafter, the wells were incubated with mAbs to AREG, either AR37 or AR558. Following incubation for 2 h with an anti-mouse antibody conjugated to horseradish peroxidase and incubation with ABTS (2,2’-azino-bis(3-ethylbenzothiazoline-6-sulfonic acid)), signals were determined using an ELISA reader. Shown are average signals and standard deviations (bars) of triplicates. **C** Surface plasmon resonance was employed to monitor association and dissociation of ligand-mAb AR37 complexes. The mAb was immobilized on a CM5 chip. After activation, mAb AR37 (2.5 μg/ml) was injected for 5 min to reach 1300 RU. The association of AREG with AR37 was monitored by injecting different concentrations of AREG for 5 min at a flow rate of 30 μl/min. The dissociation of AREG from AR37 was investigated by stopping injection of AREG. We similarly used other growth factors, such as human and murine AREG, human HB-EGF, and additional factors (see Table 1). The presented sensograms were fitted to a steady-state model. Each experiment was repeated at least twice. **D** HeLa cells were incubated without or with human AREG (100 ng/ml) and increasing concentrations of mAb AR37. Thereafter, cell extracts were resolved using electrophoresis and immunoblotting that used the indicated antibodies.

When combined with chemotherapy, a humanized antibody against VEGF, bevacizumab, improved progression-free survival of patients with therapy-resistant OvCa, which led to clinical approval of the treatment [14]. We assumed that EGFR ligands, especially AREG and EREG, which are frequently co-expressed in carcinomas [15], might offer similar treatment opportunities. ELISA-based analysis of ascites fluids, which we collected from 78 patients with OvCa who were pre-treated with chemotherapy, revealed that both VEGF and AREG were abundantly expressed in most patients, but EREG was detectable only in very few samples (Fig. 1A). This observation extended our previous analyses [5], and they motivated an attempt to target AREG in OvCa models. Due to the emerging functions of AREG in orchestrating immunity, inflammation, and tissue repair [16], the use of immunocompromised animal models may not reflect the true potential of blocking AREG. Hence, we aimed at establishing an animal model that permits blocking the murine form of AREG in the context of an intact immune system. To begin with, we expressed the EGF-like domain of AREG as a fusion protein, linked to thioredoxin [17]. Because we were interested in antibodies that recognize both the human and the rodent forms of AREG, the human growth factor was used for vaccination of mice lacking expression of amphiregulin (AREG^−^^/−^ mice). Standard hybridoma techniques were used when generating AREG-blocking monoclonal antibodies (mAbs).

One of the selected mAbs, AR37, recognized both the human and the murine forms of AREG (Fig. 1B). For reference, we used mAb558, which is specific to human AREG (hAREG). Because weak signals were detected when testing another EGFR ligand, HB-EGF, we employed the more sensitive surface plasmon resonance assay. As expected, mAREG and hAREG displayed nanomolar range binding constants, but the affinity toward HB-EGF was approximately 100-fold lower (Fig. 1C and Table 1). Next, we verified that mAb AR37 can block AREG-induced activation of EGFR (Fig. 1D). In conclusion, in similarity to VEGF, AREG is highly abundant in abdominal fluids of patients with OvCa. By immunizing AREG-knockout animals, we were able to select a neutralizing mAb.

**Table 1:** Binding of the selected antibody (mAb37) to EGF-like factors.

Ligand	KD (molar)
Human AREG	3.24E–09
Murine AREG	6.40E–09
Human HB-EGF	2.46E–07
Murine HB-EGF	ND
Human EGF	ND
Murine EGF	ND
Human Epiregulin	ND
Human TGF alfa	ND
Murine TGF alfa	ND
Human Epigen	ND
Human NRG1	ND
Murine NRG1	ND

Surface plasmon resonance was employed to monitor association and dissociation of ligand-mAb AR37 complexes (see Fig. 1C). Sensograms were fitted to a steady-state model.

*ND* non-detectable.

### Blocking AREG inhibits a murine animal model of OvCa

**Fig. 2:**
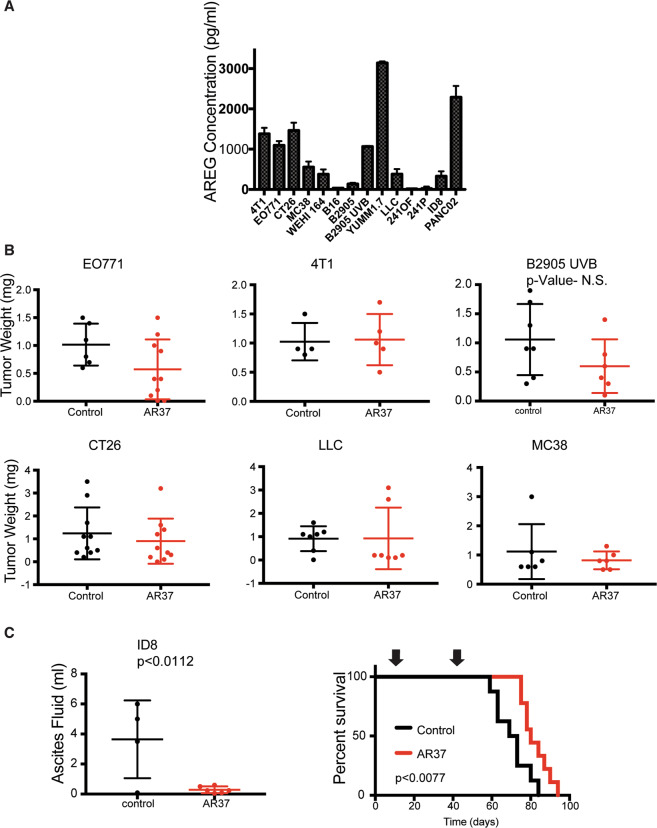
AREG is secreted by murine tumor cell lines, including ID8 ovarian cells, which are inhibited by antibody AR37. **A** Amphiregulin secretion by the indicated murine cancer cell lines was assayed in duplicates using media conditioned over 4 days and ELISA. **B** Female C57/Black or Balb/c mice were inoculated subcutaneously with the following cancer lines: B2905 UVB (5 × 10^5^ cells, SC), EO771 (2 × 10^5^ cells, into the mammary fat pad), 4T1 (2.5 × 10^5^ cells, fat pad), CT26 (5 × 10^5^ cells, SC), LLC (5 × 10^5^ cells, SC), and MC38 cells (5 × 10^5^ cells, SC). When tumors became palpable, mice were randomized into two groups, which were treated intraperitoneally with control or with AR37 mAb (0.2 mg/mouse, twice a week), and tumor weights were determined. **C** C57/Black female mice were injected intraperitoneally with ID8 cells (5 × 10^6^ per mouse). Left panel: one group of six mice was treated twice a week with AR37 (0.2 mg/injection) and another group of four mice was left untreated. Ascites fluids were collected on day 75 from both groups and their volumes are presented. Right panel: one group of nine mice was treated with AR37 and another group was left untreated. Treatments started on day 10 and lasted until day 45 (arrows). Shown are animal survival curves. This experiment was repeated four times.

The availability of AR37 enabled us to examine several syngeneic xenograft models. In the first step, we assayed media conditioned by 14 murine cancer cell lines, and found that most lines secreted large amounts of AREG (Fig. 2A). Next, we selected six non-OvCa cell lines (B2095 melanoma, EO771 and 4T1 breast, CT26 and MC38 colon and LLC lung cancer) for xenograft studies: when tumors became palpable, mice were treated intraperitoneally with saline or with AR37. Notably, no model was affected by AR37 and no adverse effects were detected (Fig. 2B). Next, we tested the ID8 model, which is the most widely used murine model of OvCa [18]. In our experiments, ID8 cells were implanted in female mice, and 10 days later mice were treated with mAb AR37. Following 11 injections, mice were left untreated, but we kept monitoring the circumference of the abdomen. Mice were sacrificed when body weight reached 25 g, or when abdomen’s perimeter reached 8 cm. Figure 2C presents the volumes of abdominal fluids, along with Kaplan–Meier animal survival curves. Because clear differences were observed, in favor of the tested monotherapy, we next focused on the underlying mechanism of action.

### Blocking AREG by means of mAb AR37 inhibits proliferation and migration of ID8 cells

To resolve mechanisms of action of mAb AR37, we examined the effects on proliferation and migration of ID8 cells. Incubation with the murine form of AREG, along with increasing concentrations of the antibody, confirmed dose-dependent inhibition of EGFR phosphorylation and ERK activation (Supplementary Fig. S1A). Because ID8 cells secrete AREG (Fig. 2A), we assumed that AR37 would inhibit DNA synthesis. The respective assay utilized a radioactive nucleoside, and the results indicated dose-dependent inhibition of incorporation into DNA (Supplementary Fig. S1B). Next, we addressed cell migration. As expected, AREG enhanced migration of ID8 cells (Supplementary Fig. S1C) and the antibody inhibited migration (Supplementary Fig. S1D). Assuming that additional EGF-like growth factors are secreted by ID8 cells, we tested the effect of a decoy, TRAP-Fc, comprising the extracellular domain of EGFR, fused to the ectodomain of ERBB4 [19]. The magnitude of the inhibitory effect we observed with the decoy was similar to the effect of AR37, implying that autocrine AREG acts as a major driver of ID8 cell migration. In summary, by blocking AREG, AR37 can inhibit both migration and proliferation of murine ovarian surface epithelial cells.

### Knockout of *areg* reduces tumorigenicity of ID8 cells in a syngeneic animal model

**Fig. 3:**
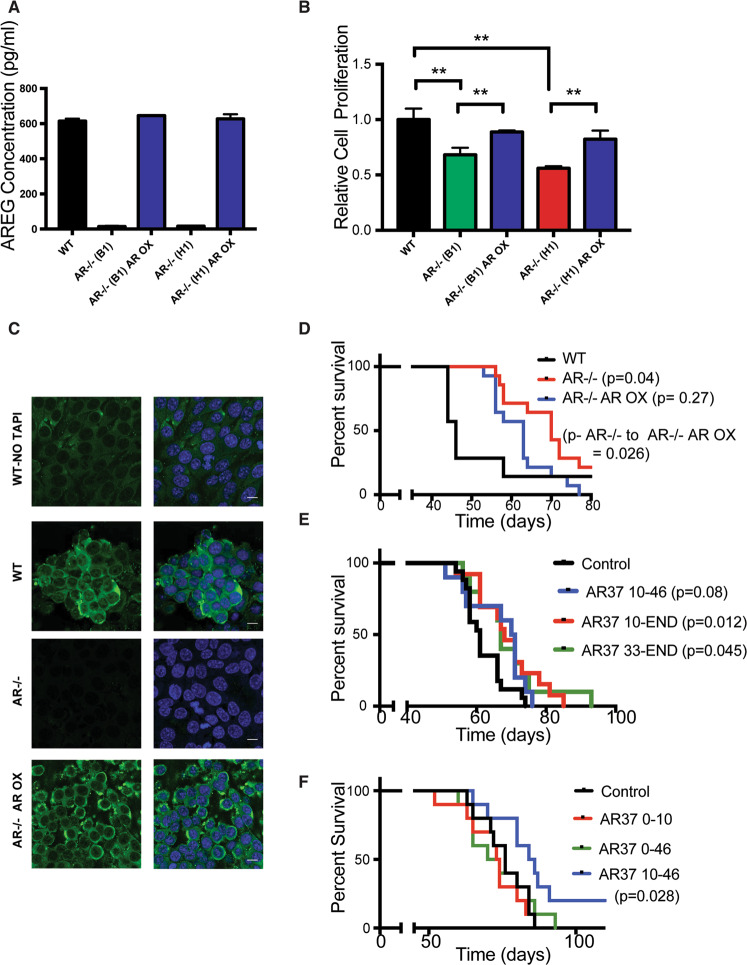
Knockout of AREG inhibits proliferation of ID8 cells and reduces their malignancy in animals. **A** AREG secretion by wildtype ID8 cells and two sublines, in which we inactivated the AREG gene. Also presented are the respective two rescue sublines (denoted AR^−/−^ (B1) AR OX and AR^−/^^−^ (H1) AR OX). All lines were maintained for 4 days in low serum (1%) medium and, thereafter, the media were collected for ELISA. Shown are mean ± SD (bars) of triplicates. **B** The cell lines from A were seeded in 96-well plates (300 cells/well). Twelve hours later, the media were replaced by serum-free media and cells were allowed to grow for 7 days. Cell viability was determined using the MTT assay (mean ± SD of three independent experiments performed with triplicates). **C** The indicated lines grown on coverslips (0.2 × 10^6^ per assay) were pre-treated (or untreated, upper row) with TAPI and then fixed in formaldehyde. Thereafter, cells were incubated overnight with an anti-AREG primary antibody (green), followed by a secondary, FITC-conjugated antibody. DAPI (blue) was used to stain nuclei. Bars, 10 μm. **D** C57/Black mice were injected intraperitoneally with ID8 cells (5 × 10^6^; 7 mice), AREG-knockout cells (AR^−/−^; 14 mice), or the rescued cells (AR^−/−^ AR OX; 14 mice, clone B1). The following median survival times were recorded: ID8: 46 days, AR^−/−^: 70 days, and the rescued cells: 63 days. The calculated *p* value comparing AR^−/−^ tumors and the AREG rescued tumors (AR^−/−^ AR OX) is indicated (*p* < 0.026). **E** C57/Black female mice were injected intraperitoneally with ID8 cells (5 × 10^6^ per mouse). The control group included 18 mice. Ten mice were treated twice a week with AR37 from day 10 till day 46 (AR37 10-46), 13 mice were treated from day 10 till the end (AR37 10-END), and 10 mice from day 33 till the end (AR37 33-END). **F** C57/Black mice were injected as in **E**. Groups of ten mice each were treated as follows: a control group, a group treated twice a week with AR37 from day 1 till day 10 (AR37 0–10), mice that were treated from day 1 till day 46 (AR37 0–46), and a group treated from day 10 till day 46 (AR37 10–46). Median survival times: ID8 control: 76 days; AR37 0–10: 73.5 days; AR37 0–46: 72 days, and AR37 10–46: 85 days. Only the latter group was significantly inhibited by AR37 (*p* < 0.028).

Silencing *areg* might disclose the maximal potential of targeting autocrine AREG. Hence, we applied the clustered regularly interspaced short palindromic repeats (CRISPR/Cas9) method. Following genomic sequencing, two *areg*-knockout clones of ID8 cells were selected (Supplementary Fig. S2A, B) and ELISA confirmed absence of AREG secretion (Fig. 3A). To verify this, we rescued AREG using plasmid overexpression (clones denoted AR^−/−^ AR OX), and confirmed that gene ablation reduced basal proliferation rates while the rescue partly restored the original phenotype (Fig. 3B). Next, we applied immunofluorescence to identify the subcellular localization of AREG. Notably, the precursor form of AREG, pro-AREG, undergoes cleavage by a surface proteinase, ADAM17 (a dis-integrin and metalloproteinase), which generates the soluble form [20]. Hence, we compared AREG expression in the presence or absence of TNF-alpha processing inhibitor (TAPI), an inhibitor of ADAM family enzymes. The results showed only weak staining in the absence of TAPI, but the signal was strongly enhanced in the presence of the inhibitor (Fig. 3C). Likewise, no staining was observed in the KO cells, but the rescued cells displayed enhanced staining, which decorated the cell surface and periphery.

Consistent with the ability of AR37 to inhibit tumorigenic growth, inactivation of *areg* markedly prolonged survival in the syngeneic model and *areg* rescue partly restored the aggressive phenotype (Fig. 3D). Notably, in comparison with mice bearing ID8 tumors that were treated with AR37, the anti-cancer effect of *areg*’s genetic ablation was much larger (compare Figs. 2C and 3D). This proposed that the timing or duration of treatment were sub-optimal. Hence, we tested four different therapeutic regimens: (i) treatments that started on the day of ID8 cell inoculation, (ii) a 5-week-long treatment that started on day 10 after inoculation, (iii) a longer treatment that started on day 33, or (iv) a similar treatment that started on day 10. The results presented in Fig. 3E, F indicated that the most effective protocol was the one that started earlier (day 10), and continued till the end of the experiment (*p* = 0.012). In conclusion, prolonged inhibition of AREG is needed for effective antibody-mediated retardation of the ID8 cancer model in immune-competent animals.

### Immunotherapy of an OvCa animal model using an anti-AREG antibody is safe and blocks an autocrine loop

To explore potential adverse effects of AR37, we injected ID8 cells into the peritoneum of 18 female C57/Black mice and 10 days later we randomized all animals to two groups: one group was treated with AR37, while the other remained untreated. A third group underwent no engraftment or treatment, but body weight, fur texture, and general behavior of this and the other groups were followed. We noted no overt effects on body mass and overall animal appearance. The average weight of each group is presented in Supplementary Fig. S3A. Evidently, no differences were noted till day 45, and only minor differences, especially a small weight gain in the untreated tumor-bearing group, were noted. In conclusion, treatment with AR37 was associated with no significant adverse effects, although the mAb binds with many murine tissues.

**Fig. 4:**
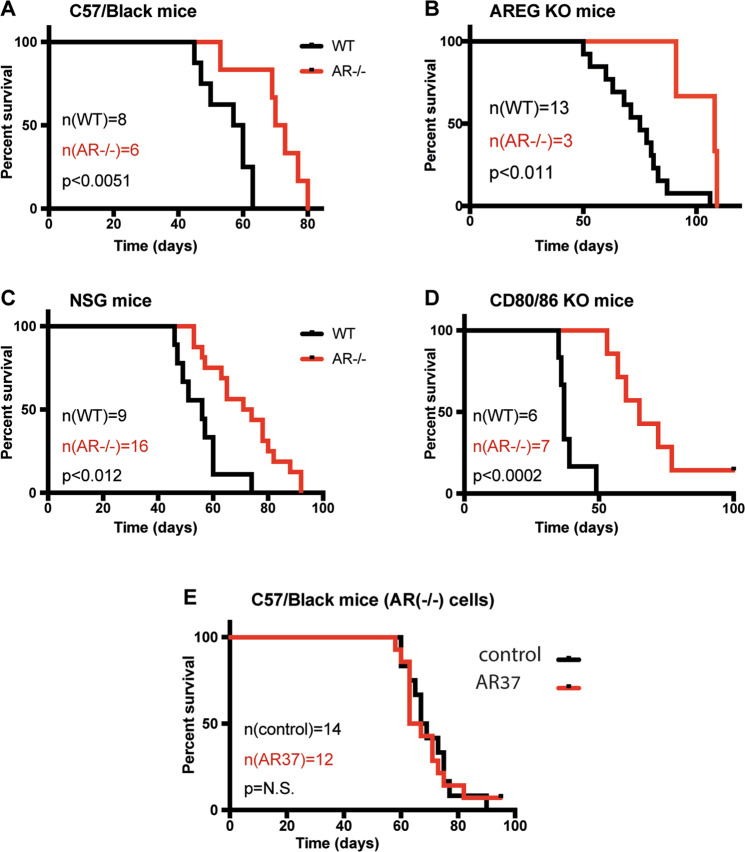
Immunotherapy of an OvCa model using AR37 involves tumor cell intrinsic mechanisms. All experiments, but **B** and **C**, were repeated at least twice. **A** ID8 (WT) and ID8 AR^−/−^ cells were injected into eight and six C57/Black mice, respectively. Median survival times: 58.5 days (WT) and 71.5 days (AR^−/−^). **B** Twenty-four AREG-knockout C57/Black mice were injected with WT ID8 cells, and 10 mice were injected with AR^−/−^ cells. The respective tumor take rates were 54% (13 mice) and 30% (3 mice). Median survival times: control, 75 days; ID8 AR^−/−^ cells: 105 days. **C** Nine NSG mice were injected with wildtype ID8 cells and 16 were injected with AR^−/−^ cells. Median survival times: control, 56 days; ID8 AR^−^^/−^ cells: 72.5 days. **D** Six CD80/86 mice were injected with WT cells and 7 mice were injected with AR^−/−^ cells. Median survival times: control, 37 days; ID8 AR^−/−^ cells: 65 days. **E** C57/Black mice were implanted with ID8 AR^−/−^ cells, and 10 days later they were randomized to two groups, which received saline or mAb AR37 (0.2 mg/injection), from day 10 onward. Median survival times were 65 days for the control group and 68 days for the AR37-treated group.

The consistent tumor-inhibitory effects of antibody AR37 might be attributed, in part, to blocking suppressive stroma-to-tumor interactions. Notably, multiple leukocyte populations, including mast cells and a subset of regulatory CD4^+^ T cells, highly express AREG [16]. To test host factors, we made use of WT and AREG-knockout mice [21]. As expected, ID8 cells expressing no AREG (ID8^−/−^) gave rise to relatively slowly growing tumors when implanted in wildtype C57/Black mice (Fig. 4A). Surprisingly, only 54% of AREG-knockout mice injected with wildtype ID8 cells displayed abdomen swelling, and this figure dropped to 30% of AREG^−/−^ mice inoculated with AREG-knockout cells. Nevertheless, ablation of *areg* in ID8 cells markedly prolonged animal survival (Fig. 4B). In conclusion, the rates of tumor take and the prolonged survival of *areg*-ablated cells imply that both host’s AREG and tumor’s AREG increase aggressiveness of ovarian cells.

The severe immunodeficient phenotype of NOD scid gamma (NSG) mice, which lack mature T, B, and NK cells, permitted us to examine involvement of the immune system. Interestingly, regardless of the immunodeficient phenotype, AREG-depleted cells still displayed markedly reduced aggressiveness in NSG mice (Fig. 4C). This observation suggested that neither lymphocytes nor NK cells are essential for the tumorigenic effect of AREG. Next, we used CD80/86 knockout mice, which display defective immunoglobulin class switching [22]. CD80 (B7-1) and CD86 (B7-2) are co-stimulatory molecules required by dendritic cells to prime naive T cells. Hence, the double KO mice lack adaptive T-cell immunity. Interestingly, ablation of AREG markedly prolonged animal survival in CD80/CD86^−/−^ mice (Fig. 4D), indicating that the mechanism underlying the oncogenic effect of AREG is independent of adaptive T-cell immunity. Taken together, these results reinforce the notion claiming that the major mechanism underlying the oncogenic effect of AREG relates to growth factor secretion by cancerous ovarian cells.

Because fibroblasts and other, non-immunological components of the microenvironment, might be involved, we asked if AR37 can prolong survival of AREG-negative ID8 cells. The results presented in Fig. 4E demonstrated that AR37 completely lost the anti-cancer effects when used to treat C57/Black mice harboring AREG^−/−^ ID8 tumors. Additional experiments observed similar results in NSG mice (Supplementary Fig. S3B), which further established the role for tumor-derived (autocrine) AREG. In the next step, we similarly tested in NSG mice both naive ID8 cells and cells overexpressing AREG. As expected, the antibody inhibited AREG-overexpressing ID8 tumors, but tumorigenesis of the naive ID8 cells was accelerated rather than inhibited by AR37 (Supplementary Fig. S3C, D). Control experiments excluded the possibility that the latter group lost expression of AREG. It is also important noting that these experiments, like all other animal tests, were repeated at least twice. Thus, although our data cannot explain the anomalous behavior of naive ID8 cells inoculated in immunocompromised NSG mice, the results we obtained with the other two groups of mice (i.e., AREG-knockout and AREG-overexpressing ID8 cells) support the observations made while using immunocompetent animals.

### Treatment of animals bearing ID8 tumors with mAb AR37 is associated with an adaptive trans-activation of EGFR by the CXCL1-CXCR2 axis

**Fig. 5:**
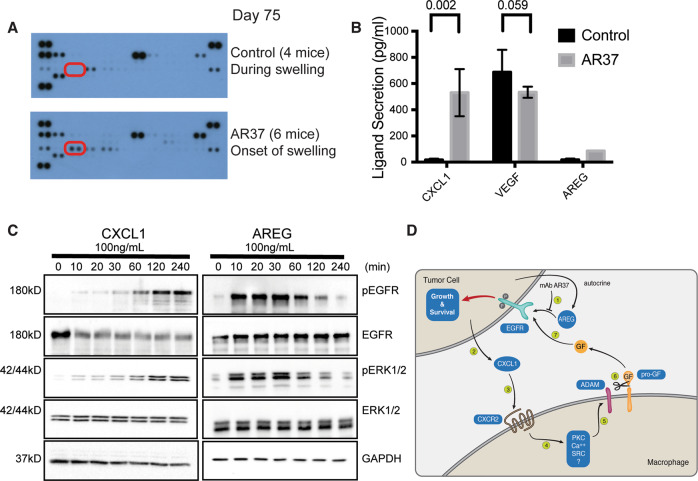
Treatment of animals bearing ID8 tumors with mAb AR37 is associated with a compensatory loop involving CXCL1. **A** ID8 cells were implanted in C57/Black mice that were untreated (four mice) or treated with mAb AR37 from day 10 till day 45 (two groups of six mice each). Ascites fluids were collected on day 75 from the untreated and from the AR37-treated mice. Pooled ascites fluids were used to overlay cytokine arrays (Proteome Profiler^TM^ Arrays). Duplicate spots corresponding to CXCL1 are highlighted. **B** Ascites fluids from control mice (*n* = 4) and from mice treated with mAb AR37 (*n* = 6) were subjected to ELISA specific to CXCL1, VEGF, and AREG. Reference standards permitted determination of exact protein concentrations. **C** Serum-starved ID8 cells were stimulated for increasing time intervals with the indicated growth factors (each at 100 ng/ml). Cleared cell extracts were resolved using electrophoresis, and immunoblotted (IB) with the indicated antibodies. **D** A schematic model of the proposed adaptive response to the AR37 antibody. In step 1, AREG binding to EGFR is blocked by AR37. In step 2, CXCL1 is secreted by the cancer cell and binds with CXCR2 expressed at the surface of macrophages (or cancer cells; step 3). Active CXCR2s stimulate a machinery involving protein kinases (step 4), which results in catalytic stimulation of ADAM family metalloproteinases (step 5). In the next step, several precursors of EGF-like growth factors (pro-GFs) are cleaved and the released factors bind and activate EGFR (step 7).

Because AREG can act as a chemoattractant [23], we assumed that AR37 treatment might alter the cytokine content of malignant ascites fluids. Hence, we used antibody arrays, which allow for the measurement of 40 murine cytokines and chemokines. Ascites fluids were sampled from untreated mice during abdomen swelling (day 75), which served as control, and from AR37-treated mice, either on day 75 (onset of abdomen swelling; sample labeled AR37-2), or when swelling was in full swing (sample labeled AR37-3). The results identified three ligands that were significantly upregulated in fluids from treated mice (Fig. 5A and Supplementary Fig. S4A): (i) the C-X-C motif chemokine ligand 1 (CXCL1), (ii) the chemokine (C-C motif) ligand 2 (CCL2/MCP1), and (iii) the interleukin-1 receptor antagonist (IL-1RA).

Notably, it has been reported that the axis connecting CXCL1 and its receptor, CXCR2, a G protein-coupled receptor, plays significant roles in mediating the communication between cancer cells and the microenvironment of gastric [24] and bladder [25] tumors. Hence, we utilized ELISA to assay CXCL1. Unlike the levels of two other ligands, CXCL1 concentrations were markedly increased in fluids derived from antibody-treated mice (Fig. 5B). In the next step, we incubated recombinant CXCL1 with naive ID8 cells and followed activation of EGFR. As predicted, CXCL1 increased phosphorylation of EGFR (as well as ERK; Supplementary Fig. S4B and Fig. 5C). However, unlike AREG, which increased phosphorylation of EGFR within a few minutes, the response to CXCL1 required longer incubation times (Fig. 5C). Because it has been reported that the CXCR2 axis is employed by IL-1β, which indirectly activates EGFR [26], we considered the possibility that the observed delayed mode of EGFR activation was due to CXCR2-mediated stimulation of a metalloproteinase, which releases into the extracellular milieu soluble forms of pro-AREG, pro-HB-EGF, and other precursors of EGFR ligands [27, 28]. Several lines of evidence are consistent with such an indirect model of EGFR activation: a single-chain antibody specific to HB-EGF (ScHB), a growth factor secreted by macrophages and other cells, inhibited the effect of CXCL1 (Supplementary Fig. S4C) and an antibody specific to the murine form of EGFR, 7A7 [29], blocked CXCL1-induced stimulation of EGFR in ID8 cells. Likewise, an inhibitor specific to CXCR2, SB225002 [30], was similarly effective and TAPI partly inhibited the action of CXCL1.

Our observations raised the possibility that surface metalloproteinases release EGFR ligands, which can replace AREG when tumors are treated with AR37. Thus, combining AR37 and CXCR2 inhibitors might have cooperative anti-OvCa effects. To further explore clinical relevance, we analyzed patient ascites fluids data collected by Lieber et al. [31]. Our analysis (Supplementary Fig. S5) implied that tumor cells co-express CXCL1 and EGFR, but expression levels of CXCR2, HB-EGF, and ADAMs are relatively low. Reciprocally, tumor-associated macrophages highly express CXCR2, HB-EGF, AREG, and ADAM proteinases, but EGFR is only weakly expressed. Similarly, T cells highly express CXCR2. This raised the possibility that tumor-derived CXCL1 attracts macrophages and T cells, which release HB-EGF and might reduce the inhibitory effects of AR37. Figure 5D presents the inferred model: In response to AR37 treatment, OvCa cells elevate expression of CXCL1, which binds to CXCR2 expressed on the surface of tumor-associated macrophages and/or tumor cells. This stimulates an intracellular cascade culminating in activation of ADAM metalloproteinases, which cleave several precursors of EGF-like ligands, thereby counteracts the cancer-inhibitory action of AR37.

### *tp53* knockout reduces AREG secretion, enhances OvCa malignancy, and confers resistance to AR37, but overexpression of AREG recovers sensitivity to the antibody

**Fig. 6:**
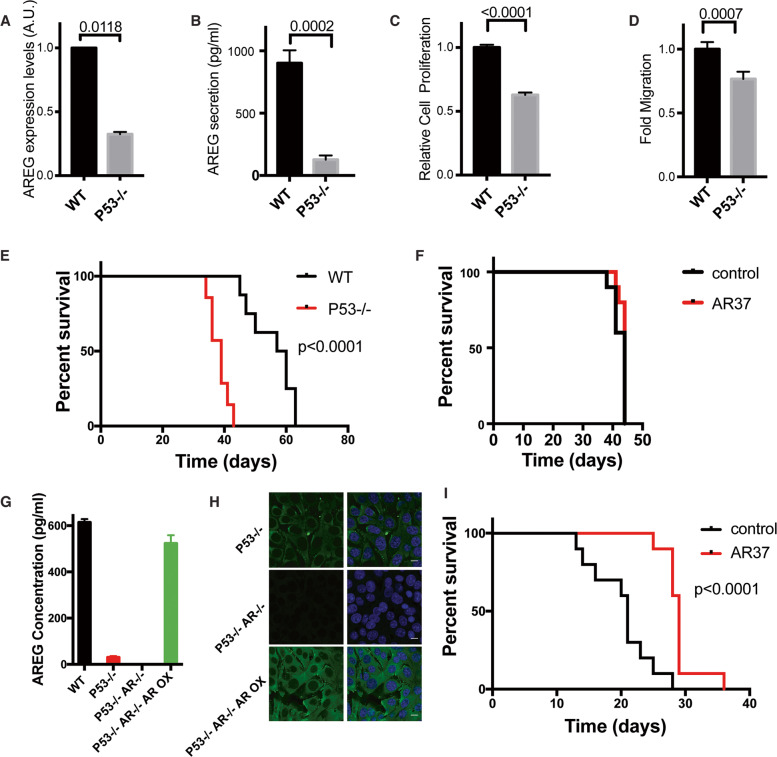
Knockout of *tp53* reduces secretion of AREG, enhances tumorigenicity, and confers resistance to mAb AR37. **A** RNA was extracted from parental ID8 and *tp53*^*−/−*^ cells and levels of AREG’s mRNA were determined in triplicates using RT-PCR. **B** The indicated cells were grown for 4 days, media were collected and AREG concentrations determined using ELISA. Shown are mean ± SD (bars) of triplicates. **C** The indicated cell lines were seeded on 96-well plates (300 cells/well). Twelve hours later, the medium was replaced with serum-free medium and the cells were incubated for 7 days, prior to determination of cell viability using the MTT assay. Shown are mean ± SD (triplicates) of three independent experiments. **D** ID8-WT or p53^−/−^ cells were seeded in the upper compartments of Transwell chambers. The lower compartment received complete medium and cells were incubated for 16 h. Paraformaldehyde was used to fix cells that reached the lower side of the filter, and crystal violet was used to stain them. For quantification of cell migration, we counted cells in four randomly selected microscope fields. **E** C57/Black female mice were injected with ID8 cells (*n* = 8 mice) or *tp53*^*−/−*^ cells (*n* = 7 mice; 5 × 10^6^ cells). Median survival times were as follows: ID8 cells: 61 days, and *tp53*^*−/−*^ cells: 38 days (*p* < 0.0001). **F** C57/Black mice were implanted with *tp53*^*−/−*^ ID8 cells as in **E**, and 10 days later they were randomized to two groups of 10 mice each: one group received saline injections, while the other was treated twice a week with mAb AR37 (0.2 mg/injection), from day 10 onward. No statistical difference was observed. **G** AREG secretion by ID8 or the indicated stable derivatives was determined as in **B** (triplicates). **H** The indicated derivatives of ID8 cells (0.2 × 10^6^) were stained for AREG as in Fig. 3C. **I** C57/Black mice were implanted with ID8 P53^−/−^ AR^−^^/−^ AR OX cells and 10 days later they were randomized to two groups of ten mice: one group received saline injections, while the other was treated twice a week with mAb AR37 (0.2 mg/injection), from day 10 onward. Survival curves are shown and the *p* value is indicated. All animal studies shown in this figure were repeated at least twice.

To explore the effect of *tp53* aberrations on the response to anti-AREG antibodies, we employed a previously described *tp53* knockout derivative of ID8 cells [32]. In line with a previous study, which found that the promoter of AREG is activated by the phosphorylated form of wildtype p53 [7], we observed markedly reduced abundance of AREG transcripts in *tp53*^*−/−*^ cells (Fig. 6A). Consistently, *tp53*^*−/−*^ cells also displayed significantly reduced secretion of AREG (Fig. 6B). Interestingly, in comparison to the parental ID8 cells, *tp53*^*−/−*^ cells displayed reduced proliferation (Fig. 6C) and migration rates (Fig. 6D), probably due to low secretion of AREG. Next, we asked if AREG expression can be restored by transfecting wildtype murine *tp53*. Transient overexpression of an ectopic p53 led to increased expression of AREG, as verified using immunofluorescence (Supplementary Fig. S6A). Concurrently, we assayed conditioned media, which indicated that the exogenous p53 upregulated secretion of AREG in both parental ID8 and *tp53*-ablated cells, in proportion to the basal levels (Supplementary Fig. S6B). In conclusion, loss of *tp53* reduced AREG expression and, conversely, regained p53 expression recovered AREG secretion.

In line with a previous report [32], when implanted in animals, *tp53*^−/−^ ID8 cells displayed significantly more robust malignant growth, relative to the parental ID8 cells (Fig. 6E), despite their relatively low migration and proliferation rates, in vitro (Fig. 6C, D). In addition, AR37 completely lost the ability to prolong animal survival when delivered to animals pre-implanted with p53-depleted cells (Fig. 6F). These observations offered the following model: as long as wildtype p53 is expressed, AREG is directly induced at the promoter level, hence permits sensitivity to antibodies like AR37. To further examine a direct link between *tp53*, AREG and sensitivity to AR37, we ablated the *areg* gene of *tp53*^*−/−*^ cells and verified, using ELISA and immunofluorescence, complete loss of secreted AREG (Fig. 6G, H). Unexpectedly, this line gained rather than lost aggressiveness in vivo (Supplementary Fig. S6C). To examine the unexpected aggressiveness of the double knockout p53^−/−^ AR^−/−^ cells, we implanted them also in another strain, CD80/86 knockout mice (Supplementary Fig. S6D). In addition, we rescued AREG expression in the double knockout cells. As expected, the new line, denoted p53^−/^^−^ AR^−/−^ AR OX, highly expressed AREG in the absence of p53 (Fig. 6G, H), and displayed remarkably short animal survival (Supplementary Fig. S6C). Interestingly, the onset of animal death was even earlier when the p53^−/−^ AR^−/−^ AR OX cells were implanted in CD80/86 knockout mice (Supplementary Fig. S6D) and the paradoxical aggressiveness of p53^−/−^ AR^−^^/−^ cells was not apparent (compare Supplementary Fig. S6C, D). Conceivably, these differences reflect immune involvement or essential roles of low AREG levels. Next, we implanted the highly aggressive p53^−/−^ AR^−/−^ AR OX cells in C57/Black animals and tested the prediction that forced AREG expression can recover sensitivity to AR37. In line with this prediction, the antibody significantly prolonged survival of the respective tumor-bearing mice (Fig. 6I). This result contrasts with the complete resistance to AR37 we observed when treating the p53^−/−^ AR^−/−^ cells (Fig. 6F). Thus, on the one hand, downregulation of AREG characterizes *tp53*-depleted OvCa cells and this associated with acquired resistance to AR37. On the other hand, forced expression of AREG in *tp53*-depleted cells recovered drug sensitivity. Hence, we concluded that AREG’s abundance is able to predict sensitivity to treatment with anti-AREG antibodies.

### High AREG predicts poorer patient prognosis and associates with ovarian tumors expressing wild-type p53

**Fig. 7:**
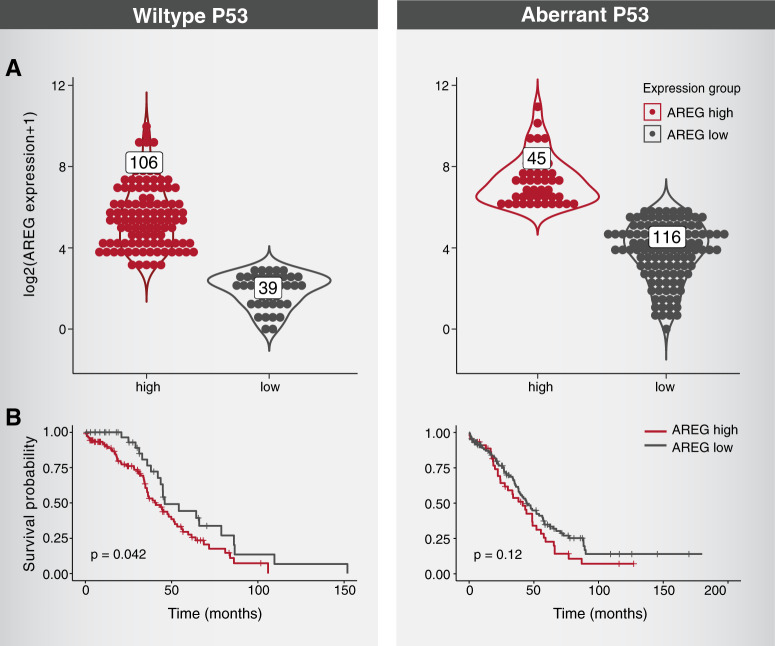
High expression of AREG associates with poorer prognosis of patients with ovarian cancer, only if tumor’s *TP53* is wild type. **A** Patients with epithelial ovarian cancer (TCGA dataset) were separated according to the status of *TP53*, wild-type (145 patients) or aberrant (161 patients), and the abundance of *AREG*’s transcript indicated. Each dot represents an individual patient. **B** Kaplan–Meier survival plots were separately generated for each of the groups shown in **A**, and the corresponding *p* values were calculated. Note that AREG expression is associated with poor prognosis only in the wildtype *TP53* group.

To examine clinical relevance, we analyzed data collected by the Cancer Genome Project (TCGA). Epithelial ovarian tumors were separated according to *TP53*’s status, either wild-type (likely a mixture of low-grade tumors; 145 patients) or aberrant (high-grade serous carcinomas (HGSC); 161 patients, see Fig. 7A). This analysis revealed that the presence of wildtype *TP53* correlates with high expression of AREG. Reciprocally, the majority of patients expressing an aberrant form of *TP53* lowly expressed AREG. These observations correlate with reduced expression of AREG in murine OvCa cells in which *tp53* was inactivated (Fig. 6A, B). Next, we asked whether AREG expression levels can predict poorer prognosis of patients with OvCa. The survival curves presented in Fig. 7B confirmed this prediction: we found that high AREG associates with poor prognosis of patients with OvCa, but this holds true only if tumors express the wildtype form of p53. These findings resonate with the differential ability of AR37 to inhibit parental ID8, unlike p53-depleted ID8 cells, which were resistant to AR37 (Fig. 6F). Taken together, the presented analyses of patient data are reminiscent of observations we made while studying an animal model of OvCa.

In summary, we report that abdominal fluids obtained from patients with OvCa frequently contain high concentrations of AREG and VEGF. Accordingly, we examined in an immune-competent animal model of OvCa both efficacy and toxicity of a novel anti-AREG strategy. Two lines of evidence supported the conclusion that targeting AREG would be beneficial for a fraction of patients: (i) knockout of the *AREG* gene strongly reduced malignancy in our model, and (ii) a new anti-AREG antibody we generated reproducibly inhibited the OvCa model. In line with on-target effects, we unraveled an adaptive regulatory loop, which can trans-activate EGFR while circumventing AREG. Importantly, our results identified AREG abundance as a predictive biomarker: high AREG confers sensitivity to the antibody, while downregulation of AREG, which is driven by loss of *tp53*, confers resistance. Because the anti-AREG antibody we generated is active and safe as monotherapy, combinations with other modalities might further enhance pharmacological efficacy.

## Discussion

In view of the failure of genomic analyses to identify druggable mutations in OvCa [2], an alternative would be targeting non-mutated proteins necessary for cell survival [33]. Several lines of previous evidence implicated AREG in tumor development (reviewed in [34]). However, one drawback of blocking non-oncogene targets like AREG is potential toxicity. Because of potential adverse effects and the emerging functions of AREG in immune processes [9], we selected a syngeneic OvCa model, ID8, and generated an antibody, AR37, specific to the murine form of AREG. Although this syngeneic model is the most commonly used in OvCa research, parental ID8 cells lack mutations in *tp53, brca1*, and *brca2*, as well as demonstrate homologous recombination competence [13]. Employing ID8 xenografts, AR37, and immunocompetent mice, we made three major observations:

1. AR37 was both safe and effective in terms of prolonging survival of mice bearing ID8 tumors. Correspondingly, ablation of *areg* inhibited malignancy.
2. In line with on-target effects, an adaptive process involving CXCL1 binding to CXCR2 was uncovered in treated animals.
3. OvCa cells expressing wildtype p53 acquire addiction to AREG: on the one hand these tumors could be inhibited by the anti-AREG antibody, and, on the other hand, deletion of p53 reduced AREG expression and conferred resistance.

Importantly, by following body weight, fur texture, and general behavior of antibody-treated mice, we concluded that treatments with AR37 are associated with no overt side effects. This observation is not surprising in light of the very mild phenotype of AREG-knockout mice [21].

By means of gene ablation and assays performed both in vivo and in vitro, our study established for the first time a functional link between the status of p53 and response to anti-AREG immunotherapy. According to previous studies, both wildtype and mutant forms of p53 regulate AREG abundance, but the underlying mechanisms differ: while mutant forms indirectly activate EGR1, which enhances AREG expression [8], the phosphorylated form of wildtype p53 directly binds with and activates the AREG promoter [7]. Our results confirmed that wildtype p53 can upregulate AREG expression and secretion, thereby permits responses to AR37. Reciprocally, depletion of p53 transformed ID8 cells to become resistant, while rescue of p53 regained both AREG expression and sensitivity to the anti-AREG antibody. These observations are critical because they identify subsets of patients with OvCa who might respond to anti-AREG strategies. For example, we predict that subsets of low-grade serous, endometrioid, mucinous, and clear-cell carcinoma, which frequently express wildtype p53, might respond to anti-AREG therapy. However, anti-AREG antibodies might be unable to control ovarian HGSC, which are characterized by frequent expression of aberrant p53 forms [35].

In summary, the use of immune-competent animals, along with an antibody recognizing the murine form of AREG, uncovered a safe, effective, and reproducible strategy that is potentially able to inhibit specific subsets of OvCa. This finding has been supported by means of CRISPR-mediated mutagenesis, which assigned to AREG a clear role in progression of OvCa. A similar approach enabled us to determine that p53 is involved in regulating sensitivity to the anti-AREG antibody. Altogether, our findings predict that future clinical applications will make use of combination treatments bringing together AR37, CXCR2 inhibitors, and selected chemotherapeutic agents.

## Materials and methods

### Cells and reagents

ID8 cells were provided by Katherine F. Roby (University of Kansas Medical Center KUMC), B2905 from Glen Merlino (NIH, Bethesda), B2905-UV (Yardena Samuels (Weizmann Institute), YUMM1.7 were from Marcus Bosenberg (Yale University), and *Trp53*^*−/−*^ ID8 cells were provided by Iain McNeish (the Imperial NIHR Biomedical Research Centre). Other cell lines were from the American Type Culture Collection (ATCC, Manassas, VA). All cell lines were recently authenticated. Growth factors were from PeproTech (Rocky Hill, NJ). Duo-set kits for ELISA were from R&D Systems (Minneapolis, MN). Antibodies were obtained from Santa-Cruz Biotechnology (EGFR), Cell Signaling Technology (ERK), Jackson Immuno Research Laboratories (HRP-conjugated anti-mouse antibody), or Millipore (GAPDH). SB225002 was purchased from BioTAG. A ScHB and the TRAP recombinant protein were generated in our lab.

### Determination of anti-tumor activities in animals

All animal studies were approved by the ethical committee of the Weizmann Institute of Science and they were repeated at least twice (except Fig. 2B and Supplementary Fig. S3D). Female athymic NCr-nude mice (6-week old) and Female C57/Black mice (6 weeks) were purchased from Harlan. Sample size was chosen to ensure adequate statistical power. Animals were included only if there was clear evidence for tumor growth. Randomization was performed once tumor progression became apparent, but the investigators were not blinded to the group allocation. Cells were injected subcutaneously into female mice (6-week old). Once tumors became palpable, mice were randomized and injected intraperitoneally twice a week with an antibody (0.2–0.3 mg per injection). Withdrawal of mice from the assay was according to one of three criteria: weight, abdomen’s circumference, or overall well-being.

### Cell migration assays

Cells were plated in the upper compartment of a 24-well Transwell tray. They were allowed to migrate through the intervening nitrocellulose membrane during 16 h of incubation. Thereafter, filters were removed, fixed in paraformaldehyde (3%), permeabilized, and stained with methyl violet. Cells growing on the bottom side of the membrane were quantified.

### Surface plasmon resonance

Surface plasmon resonance measurements were performed using a BIAcore 200 apparatus (GE Healthcare Life Sciences). Antibodies were immobilized on a CM5 chip using amine coupling chemistry. Sensograms were fitted to a steady-state model (T200 evaluation software). All experiments used at least two independent biological repeats.

### Gene knockout in vitro

The CRISPR-Cas9 system was used to create a double-stranded break next to the protospacer adjacent motif (PAM) sequence. The selected target was 21 bp long, including the PAM sequence in exon 2. The sequence was cloned into a pX458 plasmid with GFP expression. Single cell sorting was performed. Knockout was achieved by introducing a frameshift mutation due to insertion of a single nucleotide, C, in the guide sequence next to the PAM site.

### Analyses of patients’ ascites fluids and clinical datasets

The study was approved by the Ethics Committee of the Sheba Medical Center (IRB approvals 09-7409 and 09-7404). Ascites fluids were collected at the Sheba Medical Center after receiving patient consent. Patient clinical, mutational, and mRNA expression data were retrieved using the “cgdsr” package. Raw RNA-Seq data from the Sequence Read Archive (SRA) ERP021667 were re-analyzed. We performed adapter trimming and transcript-level quantification (GENCODE Human Release 29) using Salmon [36]. Differential expression analysis was performed using the “DESeq2” R package.

### Statistical analyses

All data were analyzed using the Prism Graphpad software and statistical analyses were performed using *t*-test and one- or two-way ANOVA with Tukey’s or Dunnett’s tests (*p ≤ 0.05; **p ≤ 0.01; ***p ≤ 0.001).

## Supplementary information

Supplemental Material
