# Supplementary material for: Targeting autocrine amphiregulin robustly and reproducibly inhibits ovarian cancer in a syngeneic model: roles for wildtype p53
Source: Oncogene. 2021 Apr 30;40(21):3665–79. doi: 10.1038/s41388-021-01784-8 (PMC8154589; doi:10.1038/s41388-021-01784-8)
Supplement: Supplementary file 1 — Supplemental Material [file 41388_2021_1784_MOESM1_ESM.docx]

**Legends to Supplementary Figures**


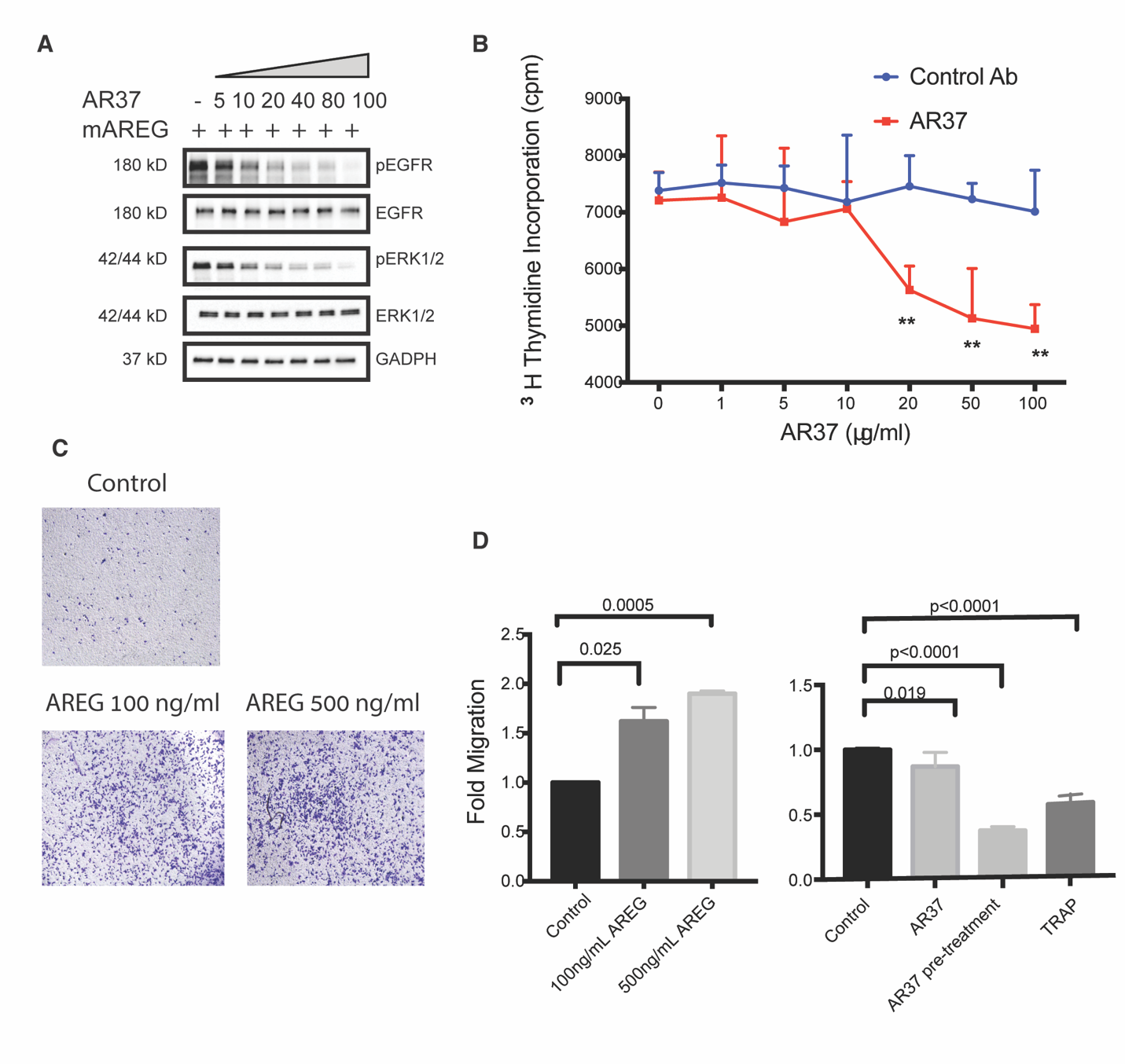


**Supplementary Figure S1: Inhibition of EGFR phosphorylation, DNA synthesis and migration of ID8 cells by mAb AR37.** (**A**) ID8 cells were incubated without or with mAREG (100 ng/ml) and increasing concentrations of AR37, an anti-AREG mAb (5, 10, 20, 40, 80 and 100 μg/ml). Thereafter, cells were lysed and cleared extracts were electrophoresed and immunoblotted with the indicated antibodies. (**B**) ID8 cells were plated onto 24-well plates at a density of 5X10^4^ cells/well. After 16 hours, the medium was replaced with fresh medium containing 1% serum, mAb AR37 (or an isotype matched control antibody) at increasing concentrations, and ^3^H-thymidine (1 µCi). The incubation was terminated 48 hours later by the addition of ice-cold trichloroacetic acid (TCA; 5%, 5 min on ice), followed by fluid collection, addition of 1N NaOH (1 ml) and incubation at 37°C for 10 minutes, followed by 1N HCL (1 ml). Radioactivity was measured in a beta-counter. Significance was assessed using two-way ANOVA followed by Sidak’s Multiple Comparison Test. Values represent means ± SEM (**, p<0.01). The assay was repeated  twice in quadruplicates. (**C**) ID8 cells were placed in the upper compartments of Transwells. The lower compartment contained mAREG, either 100 or 500 ng/ml. Migrated cells were stained with crystal violet 18 hours later. Representative fields are shown. (**D**) Left panel: Shown are quantitative assessments of the number of cells from C, which migrated to the lower chambers. Right panel: ID8 cells were treated as in C, except that no AREG was used and cells were treated with either TRAP (20 μg/ml) or mAb AR37 (20 μg/ml). AR37 was added either before or together with AREG. Results are expressed as means ± S.D. of triplicates. The experiment was repeated twice.


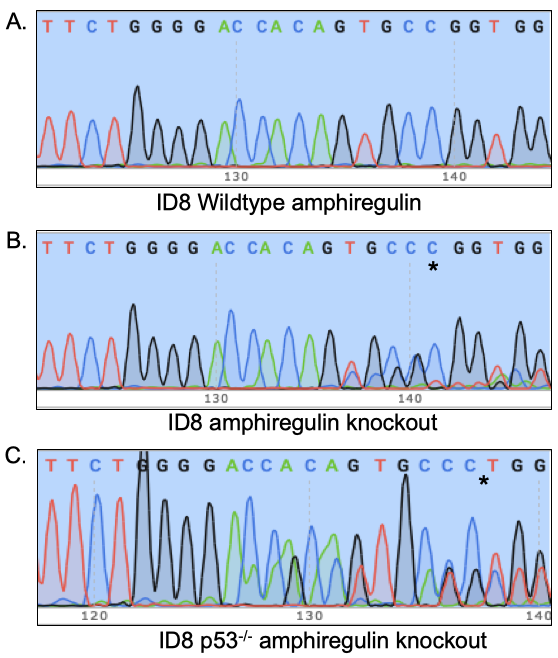


**Supplementary Figure S2: Amphiregulin knockout in ID8 cells.**  Single-guide RNAs (sgRNAs) targeting the second exon (TTCTGGGGACCACAGTGCCGGTGG) of the murine amphiregulin gene were designed and cloned into the pX458 plasmid. After transfection into ID8 cells,  we sorted GFP-positive single cells in a 96-well plate. Thereafter, cells were allowed to reach confluence and the expression level of AREG was assayed using ELISA. Sequencing analysis was performed on the genomic DNA of (**A**) wildtype and (**B**) *areg* knockout ID8 cells. An asterisk indicates the insertion site.


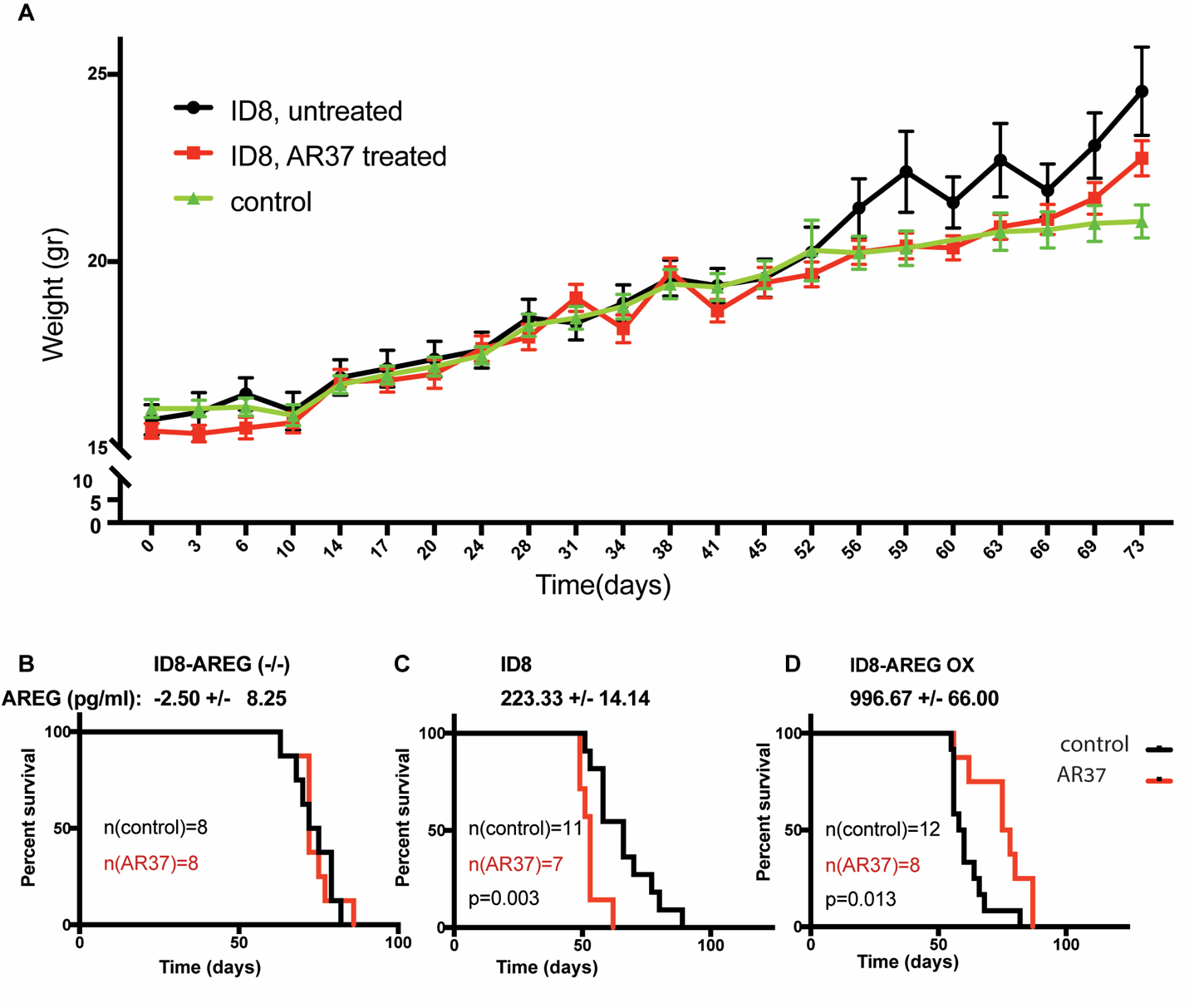


**Supplementary Figure S3: In immunocompetent mice, AR37 only slightly changes body weights, and in immunocompromised mice it inhibits AREG-overexpressing ID8 tumors.** (**A**) Two groups of 9 C57/Black mice were injected intraperitoneally with ID8 cells (5X10^6^ per animal), whereas a third group (12 mice) was left untreated (control). Ten days later, one group received treatment with AR37 (0.2 mg per injection), which was repeated twice a week until day 45. Body weight was determined twice a week. The average body weight (±S.D.) of each group is presented. (**B**-**D**) NSG mice were injected with ID8 cells (5x10^6^ per animal), which were later randomized into two groups of 7-12 animals per group. One group received saline injections (control; black lines) and the other (red lines) received mAb37 (0.3 mg/injection; twice a week). Mice were injected with either AREG-knockout ID8 cells (B) or with naive ID8 cells (C). Alternatively, mice were injected with AREG-overexpressing ID8 cells (D). ELISA was used to determine the concentration of AREG secreted by each of the three cell lines (upper row). Treatments started on day 10 and terminated on day 45. Animal survival curves are shown.


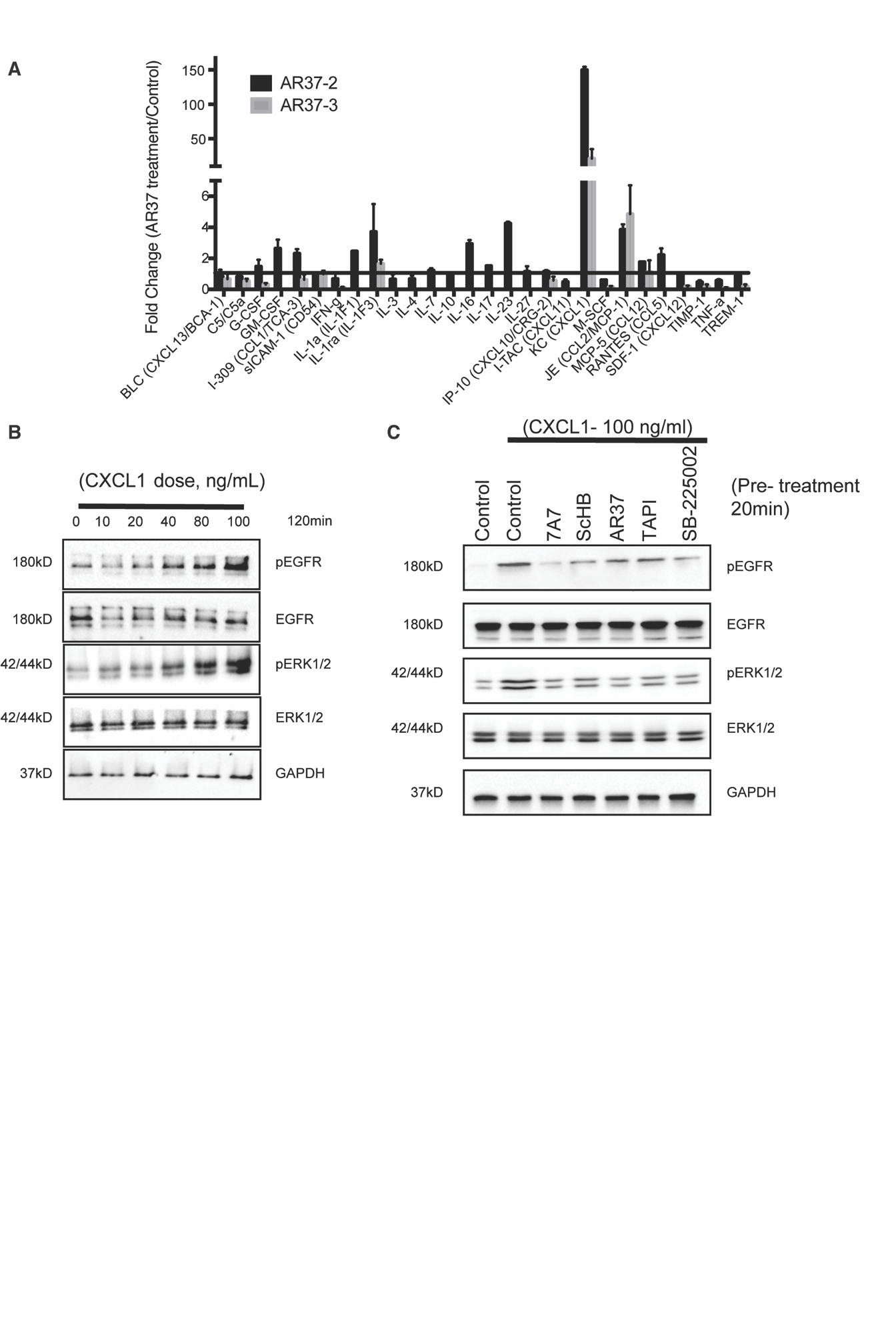


**Supplementary Figure S4: Treatment of tumor-bearing animals with mAb AR37 associates with elevated CXCL1 levels, which can activate EGFR through an autocrine route.** (**A**) ID8 cells were implanted in C57/Black mice that were untreated or treated with mAb AR37 from day 10 till day 45 (see Fig. 5A). Ascites fluids were obtained either 75 days after inoculation of tumor cells (control and AR37-2) or a week later (day 83), when the abdomen was already swollen  (AR37-3). The ascites fluids were used to overlay cytokine arrays (Mouse Cytokine Array Panel A, Proteome ProfilerTM Array; R&D Systems). The arrays were scanned, signals were normalized to control spots and expression ratios were calculated. Bars represent ranges of duplicate spots. (**B**) Sub-confluent ID8 cells growing in 6-well plates were starved overnight for serum factors and then stimulated (or not) with increasing concentrations of CXCL1. Cells were harvested 120 minutes later and processed for immunoblotting, as indicated. GAPDH was used as a loading control. (**C**) ID8 cells were pre-treated for 20 minutes with the following agents: 7A7 (an anti-murine EGFR mAb; 20 μg/ml), AR37 (30 μg/ml), single chain anti-HB-EGF antibody (ScHB; 30 μg/ml), TAPI (4 μM) or SB225002 (10 μM). Thereafter, CXCL1 (100 ng/ml) was added, and the cells were processed 120 minutes later for immunoblot analyses using the indicated antibodies.

**Supplementary Figure S5: Relative expression levels of CXCL1, CXCR2, AREG and other proteins in different cell types found in peritoneal effusion fluids (ascites) from patients with OvCa.** An RNA-Seq dataset (SRA: ERP021667) corresponding to human tumor cells found in ascites fluids, as well as abdominal tumor-associated macrophages and T cells, was re-analyzed and differential expression analysis was performed. The genes in the heatmap are organized according to their expression, from the most abundant in the cancer cells (i.e., EGFR) to the most abundant in the immune cells (i.e., CXCR2). Colors refer to cell types and expression levels.


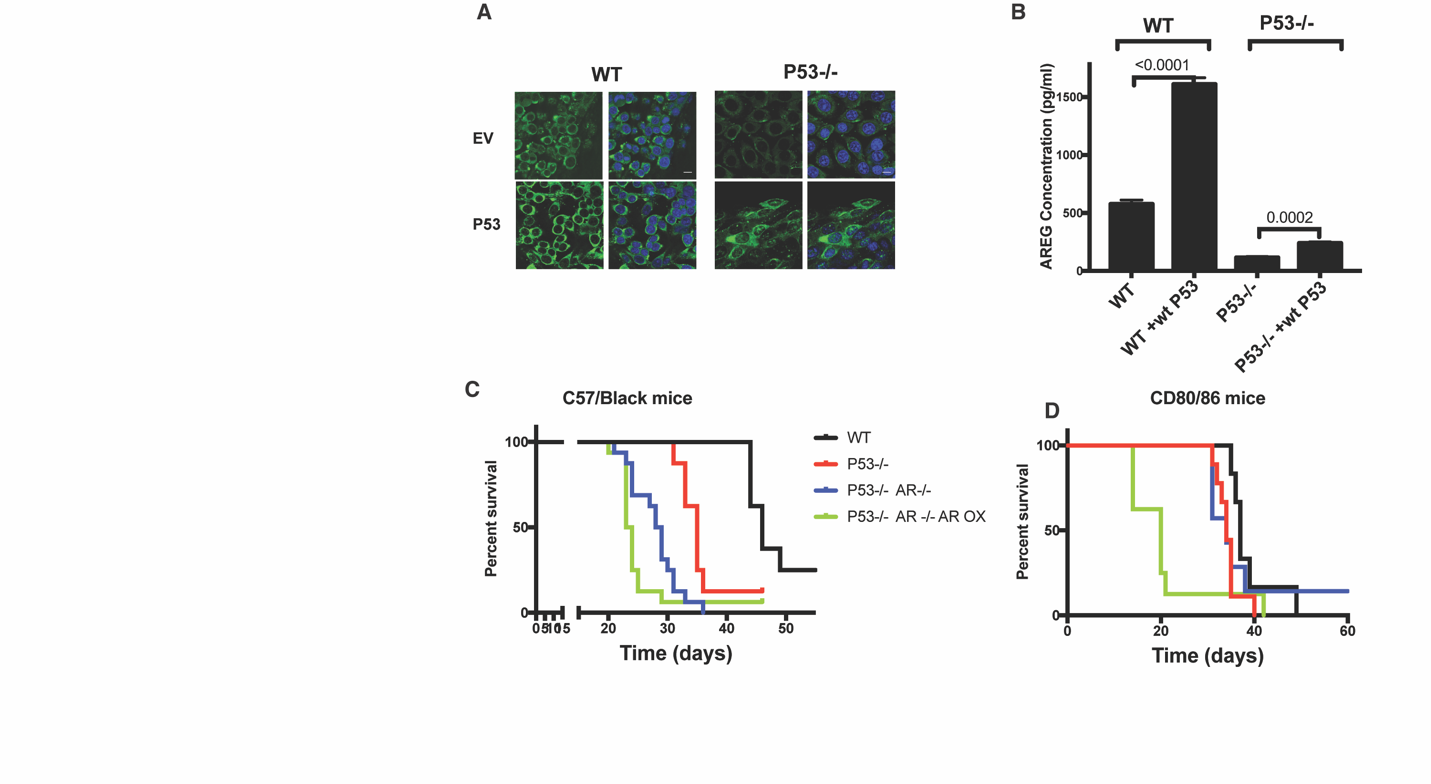


**Supplementary Figure S6: Ablation of wildtype *tp53* down-regulates expression of AREG and confers an aggressive phenotype, which is exacerbated by forced overexpression of AREG.** (**A**) Parental ID8 cells (WT) or a stable derivative lacking p53 expression, p53^-/-^ cells (0.2X10^6^), were seeded on cover slips. On the next day, cells were transfected with an empty vector or with a vector encoding the wildtype form of murine *tp53*. Twenty-four hours later, cells were treated with TAPI (20 ng/ml) for additional 24 hours and then fixed in formaldehyde (4%). Thereafter, cells were incubated overnight with an anti-AREG primary antibody (green; AR37), followed by a secondary, FITC-conjugated antibody. DAPI staining (blue) indicates locations of nuclei. Images were captured using a confocal microscope (63X magnification). Bars, 10 μM. Images are representative of three independent experiments. (**B**) Conditioned media from the cells in A were collected and AREG concentrations were determined in duplicates using an ELISA kit. The experiment was repeated twice. (**C**) Parental ID8 (WT) cells (5X10^6^), along with the indicated stable derivatives, were injected into the peritoneum of C57/Black mice. Animal survival was followed and presented in Kaplan-Meier curves. The following median survival times were observed: WT cells (8 mice): 46 days, P53^-/-^ cells (8 mice; p<0.026): 35 days, P53^-/-^ AR^-/-^ cells (16 mice; p<0.023): 28.5 days and P53^-/-^ AR^-/-^ AR OX cells (16 mice; p<0.062): 23.5 days. (**D**) CD80/86 double knockout mice were treated as in C. The following median survival times were observed: WT cells (6 mice): 37 days, P53-/- cells (9 mice; p<0.026): 34 days, P53-/- AR-/- cells (7 mice; p<0.09): 34 days, P53-/- AR-/- AR XO (8 mice; p<0.037): 20 days. Note: the animal experiments shown in C and D were repeated at least twice.
